# Marine alkaloid Monanchocidin a overcomes drug resistance by induction of autophagy and lysosomal membrane permeabilization

**DOI:** 10.18632/oncotarget.4175

**Published:** 2015-05-19

**Authors:** Sergey A. Dyshlovoy, Jessica Hauschild, Kerstin Amann, Ksenia M. Tabakmakher, Simone Venz, Reinhard Walther, Alla G. Guzii, Tatiana N. Makarieva, Larisa K. Shubina, Sergey N. Fedorov, Valentin A. Stonik, Carsten Bokemeyer, Stefan Balabanov, Friedemann Honecker, Gunhild v. Amsberg

**Affiliations:** ^1^ Department of Oncology, Hematology and Bone Marrow Transplantation with Section Pneumology, Hubertus Wald-Tumorzentrum, University Medical Center Hamburg-Eppendorf, Hamburg, Germany; ^2^ Laboratory of Marine Natural Products Chemistry, G.B. Elyakov Pacific Institute of Bioorganic Chemistry, Far-East Branch, Russian Academy of Sciences, Vladivostok, Russian Federation; ^3^ School of Natural Sciences, Far East Federal University, Vladivostok, Russian Federation; ^4^ Nephropathology Department, University Medical Center Erlangen, Erlangen, Germany; ^5^ Department of Medical Biochemistry and Molecular Biology, University of Greifswald, Greifswald, Germany; ^6^ Interfacultary Institute of Genetics and Functional Genomics, Department of Functional Genomics, University of Greifswald, Greifswald, Germany; ^7^ Division of Hematology, University Hospital Zurich, Zurich, Switzerland; ^8^ Tumor and Breast Center ZeTuP St. Gallen, St. Gallen, Switzerland

**Keywords:** Monanchocidin A, autophagy, lysosomal membrane permeabilization, germ cell tumor cells, cisplatin resistance

## Abstract

Monanchocidin A (MonA) is a novel alkaloid recently isolated from the marine sponge *Monanchora pulchra*. The compound reveals cytotoxic activity in genitourinary cancers including cisplatin-sensitive and -resistant germ cell tumor (GCT) cell lines, hormone-sensitive and castration-resistant prostate carcinoma cell lines and different bladder carcinoma cell lines. In contrast, non-malignant cells were significantly less sensitive. MonA is highly synergistic with cisplatin in GCT cells. Induction of autophagy at lower and lysosomal membrane permeabilization (LMP) at higher concentrations were identified as the dominating modes of action. Cytotoxicity and protein degradation could be inhibited by 3-methyladenine, an inhibitor of autophagy. LMP was confirmed by loss of acridine orange staining of lysosoms and by release of cathepsin B. In conclusion, MonA exerts cytotoxiс activity by mechanisms different from “classical” apoptosis, and could be a promising new compound to overcome resistance to standard therapies in genitourinary malignancies.

## INTRODUCTION

Development of resistance to standard treatment regimens is a major challenge in the course of oncological therapy. Although germ cell tumors (GCT) are highly sensitive towards platin-based cytotoxic therapies [1], 30-40% of patients with high risk metastatic disease fail to achieve a long-term response due to primary or secondary cisplatin resistance [2]. Insufficient induction of apoptosis seems to play a major role in cisplatin resistance (for review see [3, 4]). In prostate cancer, patients will inevitably succumb to the disease because of the development of resistance to treatment. Loss of efficacy of androgen deprivation therapy by up-regulation or mutation of the androgen receptor, development of resistance to docetaxel mediated by different mechanisms, e.g. up-regulation of anti-apoptotoc protein Bcl-2 [5], and resistance to abiraterone and enzalutamid mediated by androgen-receptor splice variants have been associated with disease progression [6-8]. Standard of care for patients with advanced or metastatic urothelial cancer are platin-based chemotherapy regimens. However, only 50% respond to this therapy, and more than 80% of the patients do not achieve long term remissions [9]. Thus, new drugs with different mechanisms of action to overcome resistance to standard therapies are urgently needed.

**Figure 1:**
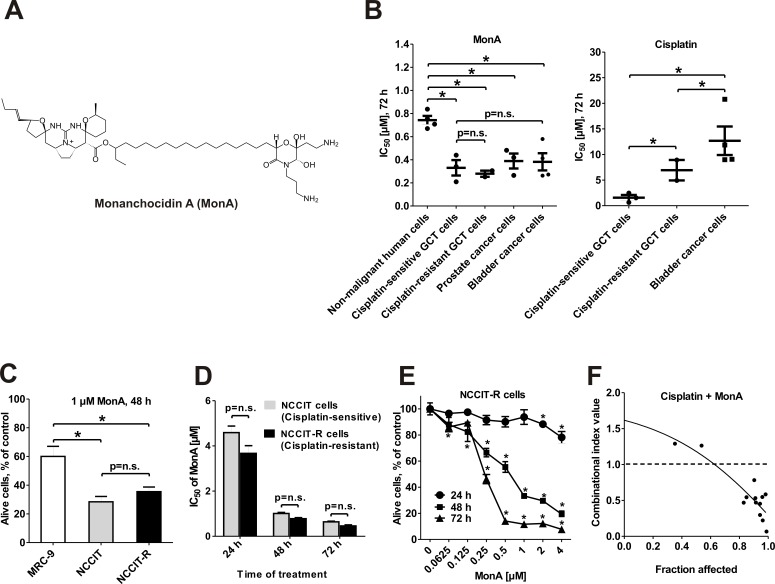
Effect of MonA on the viability of urogenital cancer cells and normal cells NCCIT and NCCIT-R cells were treated with MonA as described in Materials and Methods. **A**, The structure of Monanchocidin A (MonA). **B**, IC_50_ of MonA and cisplatin for several lines of non-malignant human cell lines as well as GCT, prostate carcinoma and bladder carcinoma cell lines after 72 h of treatment. The values are shown in Table 1. **C**, Percentage of alive cells after treatment with 1 μM of MonA, determined by the trypan blue based viability assay. **D**, IC_50_ of MonA determined by the MTT assay. **E**, Trypan blue based viability assay. NCCIT and NCCIT-R cells were treated with MonA for 48 h. Time- and dose-dependent effects of MonA on NCCIT-R cells were examined by trypan blue based viability assay. n.s.: not significant. **F**, Effect of MonA in combination with cisplatin on NCCIT-R cells, examined by the MTT assay. Cells were co-treated with different concentrations of the single substances or their combination for 48 h at a constant molar ratio. The combinational index (CI) values were calculated with CompuSyn software. The ratio of the substances is C(MonA) : C(cisplatin) = 1.2 : 10.

A relevant percentage of cytotoxic drugs have been developed on the basis of natural compounds and their derivatives [10, 11]. Remarkably, the chemical structures and variety of natural compounds found in marine organisms differ significantly from terrestrial plants and animals. Specific environmental conditions such as high pressure and salt content as well as varying pH values of the sea have been identified as possible reasons. In addition, many organisms living in the highly competitive environment of the sea have developed special defense strategies to protect themselves against enemies. Often, they produce a large variety of small molecular substances with specific bioactive characteristics. Not surprisingly, these substances moved into the focus of cancer research in the past decades. Indeed, marine drugs such as cytarabin (Ara-C), trabectedin, and eribulin are used clinically to treat different malignancies including leukemias, lymphomas, soft tissue sarcomas, and breast cancer [10-14]. Monanchocidin A (MonA, Fig. 1A) is a novel alkaloid, initially isolated from the marine sponge *Monanchora pulchra* in 2010 [15]. It reveals cytotoxic activity against THP-1, HL-60, and HeLa human cancer cell lines at micro- and nanomolar concentrations. However, the precise mechanism of anticancer action has not been elucidated to date.

In this study, we characterize the cytotoxic efficacy and the mode of action of this marine compound in human genitourinary cancer cell lines with defined levels of resistance against classical anti-tumor treatments such as androgen-deprivation, docetaxel, or cisplatin.

## RESULTS

### MonA is more active against cancer cells than against non-malignant cells

Cytotoxic activity of MonA (Fig. 1A) was evaluated in human cancer cells and non-malignant human cells by MTT assay and trypan blue assay. Remarkably, GCT, prostate cancer, and bladder cancer cell lines were found to be equally and highly sensitive to MonA (including androgen-independent PC3 and DU145 cells), while non-malignant cells were affected to a lower extend (Fig. 1B, 1C; Table 1).

**Table 1:** IC^50^ of MonA and cisplatin in non-malignant cell lines and urogenital cancer cell lines after 72 h of treatment determined with MTT assay

MonA									
	Non-malignant cell lines				Cisplatin-sensitive GCT cells			Cisplatin-resistant GCT cells	
Cell line	MRC-5	MRC-9	HEK 293T	HUVEC	NCCIT	2102EP	TCam-2	NCCIT-R	2102EP-R
IC_50_,μM	0.756	0.708	0.670	0.838	0.210	0.341	0.440	0.253	0.306

MonA							
	Prostate cancer cells			Bladder cancer cells			
Cell line	PC3	DU145	LNCaP	RT112	RT4	486p	T24
IC_50_, μM	0.420	0.482	0.265	0.263	0.58	0.273	0.413

Cisplatin									
	Cisplatin-sensitive GCT cells			Cisplatin-resistant GCT cells		Bladder cancer cells			
Cell line	NCCIT	2102EP	TCam-2	NCCIT-R	2102EP-R	RT112	RT4	486p	T24
IC_50_, μM	1.56	0.644	2.48	8.93	4.93	8.99	11.87	20.79	9.07

### MonA is equally cytotoxic against cisplatin-sensitive and -resistant GCT cells

To determine the efficacy of MonA in cisplatin-resistant GCT, the compound was examined in human GCT cell lines and cisplatin-resistant sublines. The cell lines NCCIT-R and 2102EP-R exhibit resistance to cisplatin in comparison to the original cell lines they have been generated from (Fig. 1B; Table 1) [16, 17]. MonA was equally cytotoxiс in all cancer cell lines. The level of cisplatin resistance had no impact on the efficacy of MonA (Fig. 1B; Table 1). Cytotoxicity was time- and dose-dependent. After 48 h of treatment, the IC_50_ ranged between 0.5 to 1 μM (Fig. 1D, 1E, data for NCCIT-R cells). Interestingly, activity of cisplatin could be enhanced by MonA. In fact, the combination treatment resulted in strong synergistic effects in NCCIT-R cells (Fig. 1F). Notably, the androgen-independent prostate cancer cells PC3 and DU145 were equally sensitive to MonA as GCT cells, while androgen-dependent LNCaP cells were even more sensitive (Table 1). Furthermore, bladder cancer cells, being even more resistant to cisplatin then cisplatin-resistant GCT cells, were as sensitive to MonA as other cancer cell lines (Fig. 1B; Table 1). For further investigations of the mode of action, the cisplatin-resistant NCCIT-R cell line was chosen as an example of a cell line resistant to standard chemotherapy.

### Effects of MonA on cell cycle progression and induction of programmed cell death

**Figure 2:**
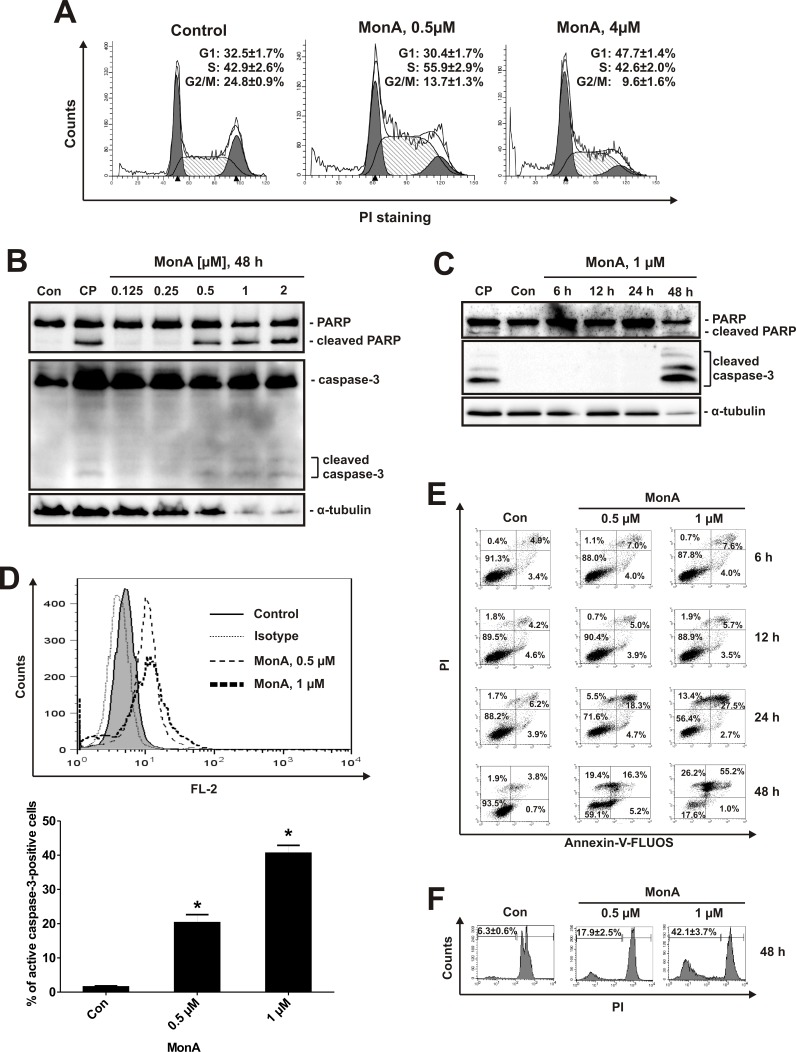
Effect of MonA on cell cycle distribution and induction of apoptosis Cells were treated with MonA as described in Materials and Methods. **A**, Cell cycle analysis of NCCIT-R cells treated with 0.5 μM and 4 μM of MonA for 24 h after cell cycle synchronization by 0.1% FBS/DMEM. Cell cycle phase distribution was analyzed and quantified using the FlowJo software. **B**, **C**, Western blotting analysis of protein extracts of NCCIT-R cells treated with MonA for 48 h with different concentrations (B), or for different timespans with 1 μM (C). NCCIT-R cells treated with 10 μM of cisplatin (CP) for 48 h were used as positive controls for induction of apoptosis. Note that the weak signal observed in the bands of the loading control α-tubulin at the highest concentrations of MonA reflects protein degradation, and not unequal protein loading of the bands. **D**, To determine the percentage of cells containing activated caspase-3, NCCIT-R cells were treated with 0.5 μM (dashed area), or 1 μM of MonA (bold dashed area), or DMSO (control, gray area), and stained with antibodies against cleaved caspase-3; or treated with DMSO and stained with isotype control (dotted area). The amount of cleaved caspase-3 positive cells was quantified using Cell Quest Pro software. The graph shows the percentage of positive cells of the total cell number in each sample. **E**, Flow cytometry analysis of NCCIT-R cells treated with MonA: Annexin-V-FITC versus PI (double staining). Apoptotic cells appearing in the right lower and upper quadrants were quantified using the Cell Quest Pro software. **F**, Cell cycle analysis of NCCIT-R cells treated with MonA for 48 h. The amount of apoptotic cells (sub-G1 population) was quantified using the Cell Quest Pro software.

After 24 h cell cycle analysis of NCCIT-R cells treated with MonA revealed a S-phase arrest at non-cytotoxic, and a G1-phase arrest at cytotoxic concentrations of MonA (Fig. 2A). Hallmarks of classical apoptosis e.g. cleaved PARP- and caspase-3 (Fig. 2B, 2C) were detected by Western blotting analyses in a time- and dose-dependent manner. Induction of caspase-3-cleavage was confirmed by flow cytometry using a PE-conjugated antibody against cleaved caspase-3 (Fig. 2D). Time- and dose-dependent phosphatidylserine externalization as well as DNA fragmentation were detected by flow cytometry (Fig. 2E, 2F). Note that a significant number of necrotic cells was observed (Fig. 2E, 2F).

### Monanchocidin A induces unselective protein degradation

**Figure 3:**
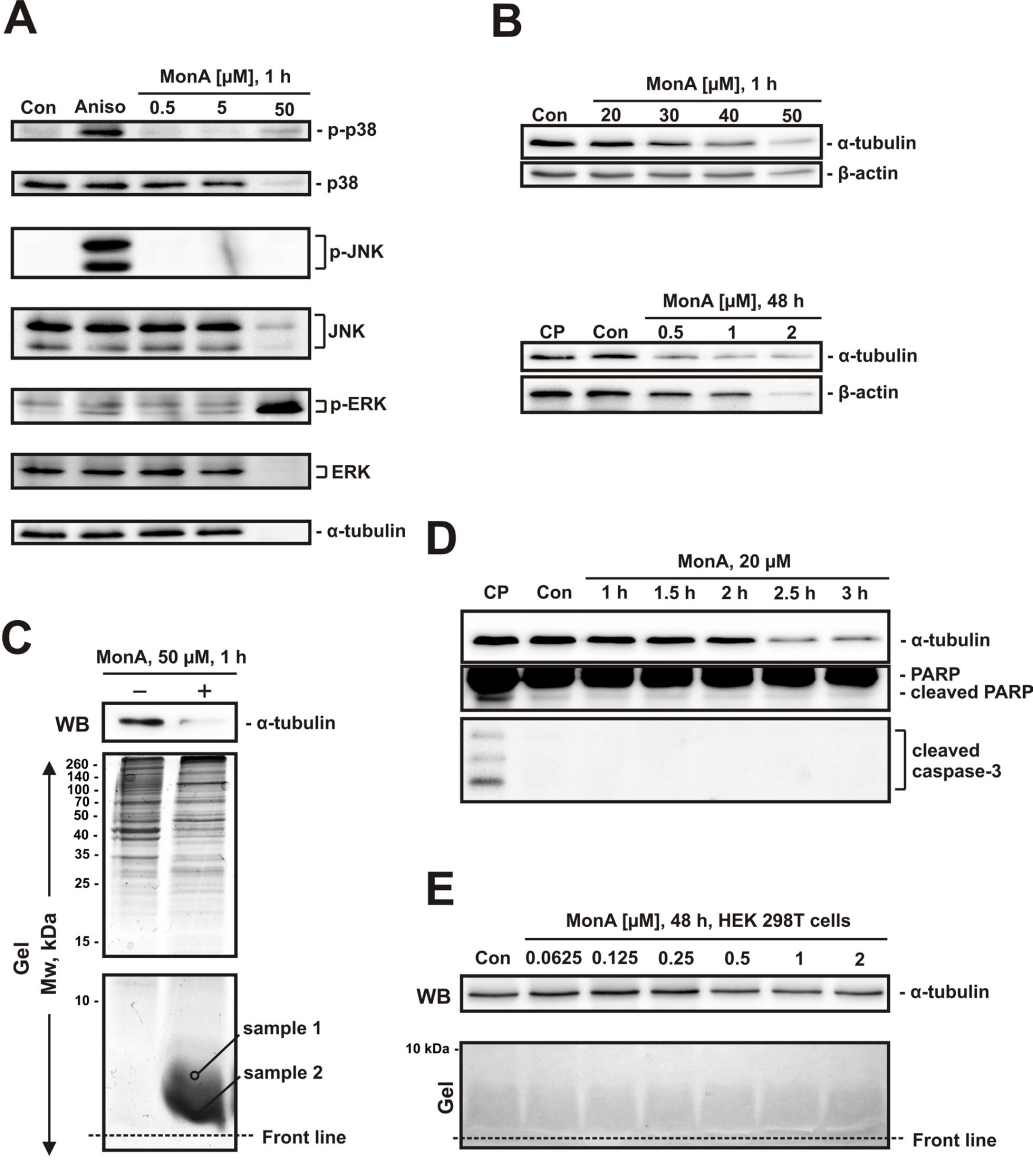
Effect of MonA on activation of MAPK and protein degradation **A**, NCCIT-R cells were analyzed after treatment with MonA for 1 h by Western blotting. Cells treated with 50 μM of anisomycin for 1 h (Aniso) were used as a positive control for MAPK activation. **B**, Protein analysis by Western blotting reveals degradation of proteins induced by MonA following short- and long-term treatment. Cells treated with 10 μM of cisplatin-treated (CP) for 48 h do not show signs of protein degradation. **C**, Analysis of NCCIT-R cells treated with 1 μM of MonA for 1 h. After treatment, cells were harvested and proteins were separated using PAGE on 15% acrylamide gels. The proteins from the upper part of the gel (containing proteins with Mw > 15 kDa) were transferred to a PVDF membrane followed by Coomassie Blue staining of the gel. The lower part of the gel was stained with Coomassie Blue without transfer stage. Two samples of the spot were randomly picked from the area between ~5 kDa and ~1 kDa, followed by trypsination and identification of peptides by tandem mass spectrometry. **D**, Analysis of NCCIT-R cells treated with 20 μM of MonA for 1-3 h. Cleavage of PARP and caspase-3 were investigated as hallmarks of apoptosis. Cells treated with 10 μM of cisplatin (CP) for 48 h were used for comparison of the effects of a classical cytotoxic agent. **E**, Analysis of non-malignant HEK 293T cells treated with 0.0625 – 1 μM of MonA for 48 h. Cells were harvested and proteins were separated using PAGE on 15% acrylamide gels. The proteins from the upper part of the gel were transferred to a PVDF, while the lower part of the gel (containing proteins with Mw < 15 kDa) was stained with Coomassie Blue without transfer stage.

Cisplatin induces cell death via mitogen-activated protein kinases (MAPK) [18]. Therefore, we examined the effect of MonA on the activity of three main MAPK. A concentration of 50 μM of MonA was used to get a more pronounced effect. Activation of p38 and ERK, but not JNK was detected (Fig. 3A). Degradation of different proteins was observed in NCCIT-R cells both after short term exposure to high doses of MonA (30 μM - 50 μM), as well as after long-term exposure to low doses of MonA (0.5 μM - 2 μM) (Fig. 3B, 3C). Surprisingly, the degradation was observed not only for MAPK (total p38, JNK, and ERK, Fig. 3A), but also for various and unrelated long-lived proteins such as α-tubulin and β-actin (Fig. 3B). In addition, significant changes in the protein patterns were observed on polyacrylamide gels stained with colloidal Coomassie Blue after PAGE. Intensive spots occurred at the lower end of the gel in the area where low molecular weight proteins are located (Fig. 3C).

We suspect that these changes are caused by protein degradation induced by the compound. To assess the composition of the spot, we performed tandem mass spectrometry analysis of two samples picked from the gel area with Mw ~5 kDa, both from the upper and lower end of the spot (Fig. 3C). Several proteins were identified. Interestingly, all of these peptides were parts of “mother” proteins of higher molecular weight (11 – 80 kDa) (see supplementary, Table S2). Therefore, we assume unselective protein degradation due to MonA exposure. Notably, this protein degradation occurs before the initiation of apoptosis. Thus, it does not depend on classical apoptosis (Fig. 3D). It is important to note, that non-malignant HEK 298T cells, treated with MonA for 48 h did not reveal any signs of protein degradation (Fig. 3E).

### Unselective protein degradation by MonA is autophagy-dependent

**Figure 4:**
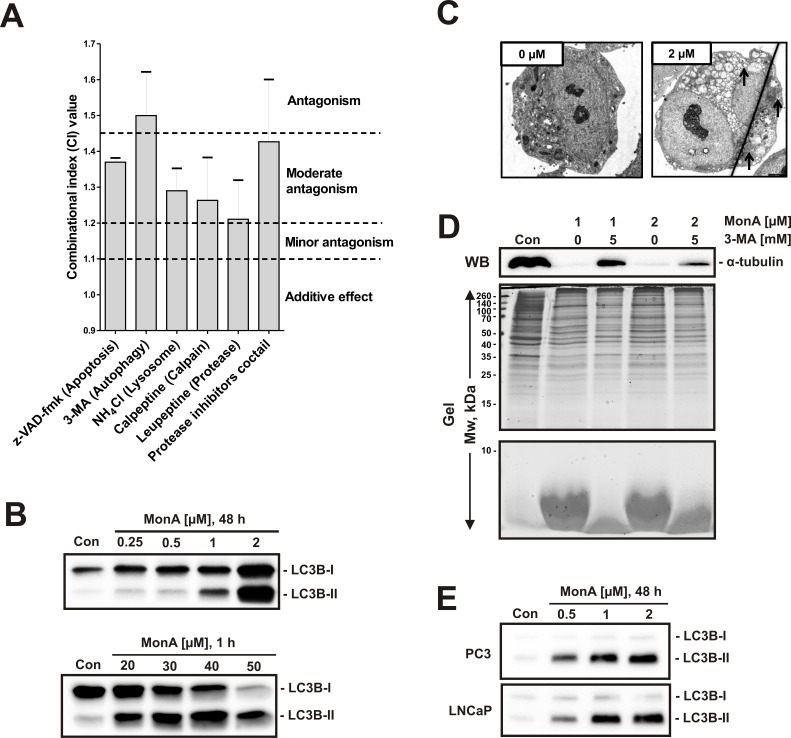
Protein degradation induced by MonA shows hallmarks of autophagy **A**, The effect of specific inhibitors on the survival of NCCIT-R cells treated with MonA was examined using the MTT assay. Co-treatment of MonA and the indicated inhibitors for 48 h was performed. The combinational index (CI) values were calculated with the CompuSyn software for the Fa (fraction affected) = 0.5. Concentration ratios of the substances used are shown in the supplementary (Table S3). **B**, Protein expression of LC3B-II, a marker of autophagy, in NCCIT-R cells following treatment with MonA at the indicated concentrations and timespans. **C**, Representative electronic microscopy pictures of NCCIT-R cells treated with 2 μM of MonA for 48 h in comparison to untreated control cells (0 μM). The number and size of autophagosomes (indicated by arrows) significantly increases after treatment. **D**, Analysis of NCCIT-R cells treated with 1 μM or 2 μM of MonA for 48 h with or without an inhibitor of autophagy, 3-MA. After treatment, cells were harvested and proteins were separated using PAGE on 15% acrylamide gels. The proteins from the upper part of the gel (containing proteins with Mw > 15 kDa) were transferred to a PVDF membrane followed by Coomassie Blue staining of the gel. The lower part of the gel was stained with Coomassie Blue without transfer step. **E**, Protein expression of LC3B-II, a marker of autophagy, in prostate cancer cells following treatment with MonA at the indicated concentrations for 48 h.

Various processes can induce protein degradation, which must be considered a lethal cellular event. Therefore we investigated the role of potential proteolytic pathways: NCCIT-R cells were pretreated with different inhibitors, including the pan-caspase inhibitor z-VAD-fmk, the autophagy inhibitor 3-methyladenine (3-MA), the lysosome inhibitor NH4Cl, the calpain inhibitor calpeptin, the protease inhibitor leupeptin, and a cocktail of several protease inhibitors (see Materials and Methods) before exposure to MonA. Potential antagonism was analyzed by the Chou-Talalay method [19, 20]. The most pronounced antagonistic effect against MonA was observed with 3-MA pretreatment. Consequently, autophagy was suspected to mediate the cytotoxic effect of MonA in NCCIT-R cells (Fig. 4A). Therefore, we analyzed expression levels of LC3B-II - a marker of autophagy [21, 22]. We found an up-regulation of LC3B-II in NCCIT-R cells both after long-term exposure to low concentrations (0.25 μM – 2 μM), and after short-term exposure to high concentrations (20 μM – 50 μM) of MonA (Fig. 4B). In addition, formation of autophagic vacuoles was observed by electronic microscopy (Fig. 4C). Finally, pre-treatment with 3-MA was able to suppress MonA induced protein degradation in Western blotting and PAGE analyses (Fig. 4D). However, pre- and co-treatment with 3-MA did not abolish protein degradation induced by MonA at concentrations > 2μM (48 h of treatment). Thus, MonA exerts a different mode of action at high concentrations and long exposure times.

The up-regulation of LC3B-II was also observed in postate cancer cells treated with MonA, suggesting that autophagy is a general mechanism underlying the cytotoxic mode of action of MonA in cancer cells (Fig. 4E).

### Classical cytotoxic agents do not induce autophagy

**Figure 5:**
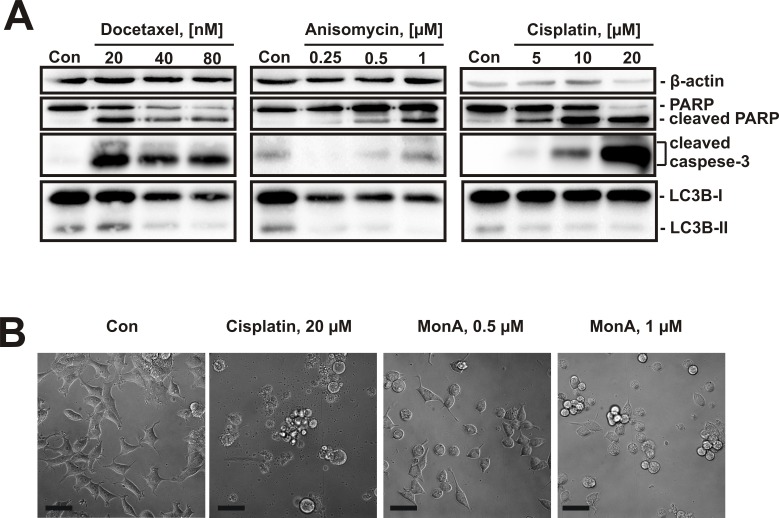
Effect of cytotoxic compounds on NCCIT-R cells **A**, NCCIT-R cells were treated with docetaxel, anisomycin, or cisplatin for 48 h. The effect on markers of apoptosis (cleavage of PARP and caspase-3) and autophagy (LC3B-II) were analyzed using Western blotting. While all show induction of apoptosis, expression of LC3B-II is not increased after treatment with these substances. **B**, Microphotographs of NCCIT-R cells treated with cisplatin or MonA for 48 h show morphological changes induced by the substances. The pictures were made at x100 magnification. Each bar represents 50 μm.

To assess whether induction of autophagy is a common phenomenon under substances known to induce classical apoptosis, we treated NCCIT-R cells with docetaxel, anisomycin, and cisplatin for 48 h. None of the drugs revealed an up-regulation of LC3B-II, while an increase of markers of apoptosis e.g. cleavage of PARP and caspase-3 were observed (Fig. 5A). In line with these observations, MonA-treated cells exhibited a different morphology (cell rounding, cytoplasm vacuolization) compared to cisplatin treated cells (cell shrinkage, membrane blebbing, apoptotic bodies formation) (Fig. 5B).

### Monanchocidin A induces lysosomal membrane permeabilization (LMP) at high concentrations

**Figure 6:**
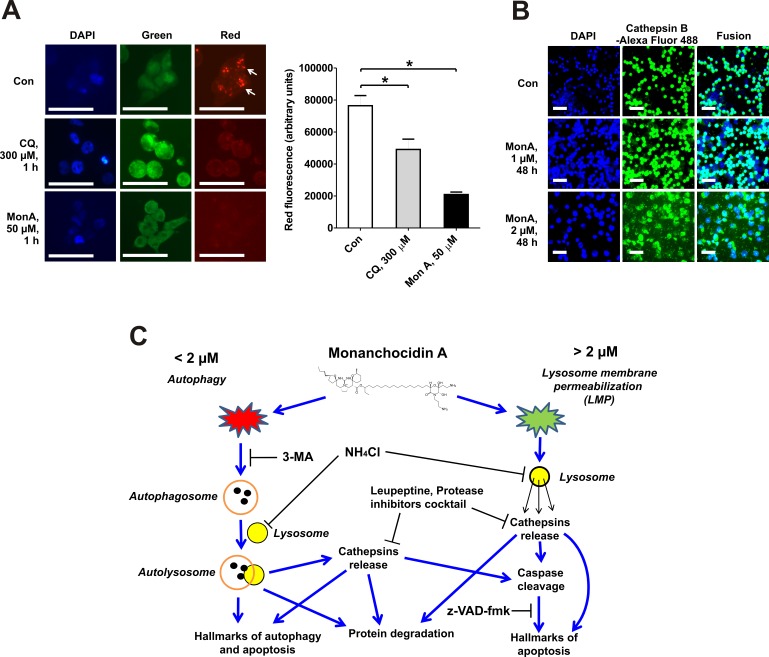
Induction of lysosomal membrane permeabilization (LMP) and model of the suggested mechanism of action of MonA **A**, NCCIT-R cells were treated for with chloroquine (CQ, positive control for lysosomal membrane permeabilization) or MonA for 1 h, stained with acridine orange and DAPI, and immediately analyzed using fluorescent microscopy. Intact lysosomes were observed as organelles emitting red fluorescence (indicated by arrows). Red fluorescence was quantified using Image J software and is graphically depicted by column bars. **B**, Cells were treated with MonA for 48 h, and immobilized on glass plates. Samples were permeabilized, blocked, labeled with a primary anti-cathepsin B antibody, followed by a secondary Alexa Fluor 488-conjugated antibody labeling, and analyzed using fluorescent microscopy. Release of cathepsin B into the extracellular space was observed in cells treated with MonA. The microphotographs (A, B) were made at x400 and x100 magnifications. Each bar represents 50 μm. **C**, Model of the suggested mechanism of action of MonA in NCCIT-R cells.

3-MA did not inhibit rapid and unspecific protein degradation in NCCIT-R cells treated with MonA at high concentrations. Permeabilization of the membranes of lysosomes, unlashing the proteolytic activity of lysosomal proteases may be underlying this observation. In contrast to other metabolic processes, LMP can start shortly after exposure to a specific compound and thus results in a rapid protein degradation [23]. In order to evaluate if this is the case for MonA, we used the lysosomotropic metachromatic fluorochrome dye acridine orange (AO) to track the integrity of lysosomes. When excited with blue light, AO emits red fluorescence at high concentrations (when localized in intact lysosomes) and green fluorescence at low concentrations (when present in the cytosol and the nucleus, for example after LMP) [24, 25]. We observed disappearance of red fluorescence coupled with increased green fluorescence, indicative for LMP induced by high concentrations of MonA (Fig. 6A). A similar effect on NCCIT-R cells was observed with chloroquine (CQ), a substance known to induce LMP [25, 26]. Further evidence for the induction of LMP was the release of cathepsin B into the extracellular space, which occurred upon treatment with MonA at concentrations of 2 μM or higher (Fig. 6B).

## DISCUSSION

MonA is a novel marine compound derived from the marine sponge *Monanchora pulchra*. MonA effectively inhibits the growth of human leukemia cell lines HL-60 and THP-1, presumably by induction of apoptosis [15]. However, to date, efficacy and mode of action of MonA in solid tumors remain elusive. We were able to show that MonA exerts high cytotoxicity in human GCT, prostate cancer, and bladder cancer cell lines. In contrast, non-malignant human cells were less sensitive to the compound. Remarkably, we found equal activity of the marine compound in castration-resistant and hormone-sensitive prostate cancer cell lines, as well as in cisplatin-resistant and -sensitive GCT cell lines, suggesting that MonA is active even if standard treatment regimens fail.

MonA induced programmed cell death and a G1- and S-phase cell cycle arrest in GCT cells. However, a more detailed examination revealed that not classical apoptosis, but autophagy and/or lysosomal membrane permeabilization are the major mechanisms leading to cell death.

Macroautophagy (referred to as autophagy in the following) is the basic cellular catabolic process of non-selective bulk degradation of damaged or long-lived proteins, organelles or/and cytoplasm by lysosomes [27-29]. It results from cellular stress, e.g. nutrient deprivation [30], and includes the formation of double-membrane vesicles (autophagosomes), cytoplasmic constituents engulfing and finally fusing with lysosomes, leading to degradation and recycling of sequestered contents [28]. However, the role of autophagy in tumor growth and suppression is controversial. In fact, opposite activity has been observed depending on different cell types and stimuli [30]. Autophagy has been found to be cytoprotective, cytotoxic, cytostatic, or even without any effect on cell survival [30-32]. However, the antitumor effects of several drugs, such as tyrosine kinases or mTOR inhibitors (for review see [28]) involve the induction of cytotoxic autophagy, also called type II programmed cell death [33].

In fact, unspecific degradation of various proteins occurred in our analyses following short-term treatment with high concentrations, or long-term treatment with low concentrations of MonA. Remarkably, protein degradation induced by long-term treatment could be inhibited by the selective autophagy inhibitor 3-MA [34]. In addition, autophagic vacuole formation after exposure to low concentrations of MonA and the up-regulation of the autophagy marker LC3B-II strongly suggest that the compound is an inductor of autophagy. In contrast, other cytotoxic substances such as docetaxel, or cisplatin, did not induce an up-regulation of LC3B-II in NCCIT-R. Thus, as expected, apoptosis, but not autophagy, is the central mechanism of action of these substances. Observation of hallmarks of apoptosis following MonA treatment is assumed to be a secondary event [23], because they appear after, but not simultaneously with protein degradation.

At high concentrations > 2 μM, the dominating mechanism of protein degradation (and therefore of cell death) of MonA was lysosomal membrane permeabilization (LMP). During LMP, cathepsins and other hydrolases are released from the lysosomal lumen into the cytosol, leading to unselective degradation of cellular components [25]. LMP is a potentially lethal event, because the presence of lysosomal proteases in the cytosol causes digestion of vital proteins and the activation of caspases and other hydrolases [35, 36]. However, cell death resulting from massive LMP can also show necrosis-like features and be caspase-independent [25, 37]. In our experiments, we found loss of lysosomal integrity as well as release of cathepsin B into extracellular space in cells treated with ≥ 2 μM MonA, supporting the idea of LMP induction.

Inhibitors of lysosomal activity and proteases were able to inhibit the cytotoxic effect of MonA to a certain degree. This also suggests that autophagy and induction of LMP are relevant for MonA activity, as both processes require active lysosoms and cathepsins. The moderate antagonistic effect of the pan-caspase inhibitor z-VAD-fmk indicates caspases activation, most likely as a rather late event. Caspase activation could result from direct cleavage of caspases by cathepsins, or be an indirect down-stream effect of autophagy and/or LMP. It should be noted that z-VAD-fmk can also inhibit other cysteine proteases including several cathepsins [38], which - at least in part - explains the antagonistic effect of z-VAD-fmk in MonA-treated cells.

Interestingly, induction of autophagy as the mechanism underlying cytotoxicity has never been reported before in GCT cells, while its induction in prostate cancer and bladder cancer cells is a known and well characterized phenomenon. It is induced by a number of anticancer drugs such as rapamycin, everolimus, metformin, and neuregulin (for review see [33, 39]). In our experiments, we have observed the induction of autophagy in both GCT and prostate cancer cells treated with MonA. As the mechanism of action of MonA strongly differs from standard agents used to treat these tumors [40], MonA might be a promising new substance capable of overcoming resistance against currently applied therapies [23, 33]. In addition, MonA may be able to enhance the activity of cisplatin in GCT cells, as synergistic effects were observed when cisplatin and MonA were combined in NCCIT-R cells. This is in line with the previous findings that LMP can promote and increase induction of apoptosis [35].

In conclusion, MonA is a highly effective marine compound, which holds promise of therapeutic activity in genitourinary cancers even if standard therapies fail. This is most likely due the distinct different mechanism of action when compared to standard chemotherapies, namely drug-induced autophagy and LMP.

## MATERIALS AND METHODS

### Reagents and antibodies

The marine alkaloid monanchocidin A (MonA) was isolated from the marine sponge *Monanchora pulchra* as previously described [15]. Anisomycin, docetaxel (10 mg/ml) and cisplatin (cis-diamminedichloroplatinum (II), 1 mg/ml) were purchased from NeoCorp (Weilheim, Germany), acridine orange and calpeptin from Sigma (Taufkirchen, Germany), MG-132 from Calbiochem (Darmstadt, Germany), NH_4_Cl and Coomassie brilliant blue G 250 from Carl Roth (Karlsruhe, Germany), 3-methyladenine and z-VAD(OMe)-fmk (referred here as z-VAD-fmk) from Enzo Life Sciences (Farmingdale, NY, USA), leupeptin from Serva (Heidelberg, Germany), protease inhibitors cocktail (cOmplete Mini EDTA-free) from Roche (Munnheim, Germany). Primary and secondary antibodies used are listed in the supplementary.

### Cell lines and culture conditions

The human prostate cancer cell lines PC3 (docetaxel resistant, androgen-independent), DU145 (docetaxel sensitive, androgen-independent), LNCaP (docetaxel sensitive, androgen-dependent) [41, 42], human bladder cancer cell lines RT112, RT4, 486p, T24, human embryonic kidney cell line HEK 293T, human embilical vascular endothelium cell line HUVEC, as well as human fibroblast cell lines MRC-5 and MRC-9 were obtained from ATCC (Manassas, VA, USA). The human germ cell tumor cancer cell line NCCIT was obtained from DSMZ (Braunschweig, Germany). TCam-2 and 2102EP cells were kindly provided by Prof. L. Looijenga (Rotterdam, The Netherlands). The cisplatin-resistant sublines NCCIT-R and 2102EP-R have been generated as reported before [16, 17]. Cells were cultured according to the manufacturers instructions (culture conditions are described in the supplementary). Cells were continuously kept in culture for a maximum of 3 months, and were routinely inspected microscopically for stable phenotype and regularly checked for contamination with mycoplasma. Cell line authentication was performed by DSMZ (Braunschweig, Germany) using highly polymorphic short tandem repeat loci.

### *In vitro* MTT-based drug sensitivity assay

The *in vitro* cytotoxicity of individual substances and drug combinations was evaluated using the MTT (3-(4,5-dimethylthiazol-2-yl)-2,5-diphenyltetrazolium bromide) assay, which was performed as previously described [43].

### Examination of synergistic/antagonistic effect of drug combination

Determination of synergistic or antagonistic drug effects in combination assays was performed using the Chou-Talalay method [20]. Data were generated by MTT assay. The combinational index (CI) was calculated for the constant drugs ratio with the CompuSyn v.1.0. software (ComboSyn, Inc., Paramus, NJ, USA). Synergism is defined as a CI < 1, whereas antagonism is defined by a CI > 1. The MTT assay was used to examine the combination of MonA at the IC_50_ with defined inhibitors of autophagy or LMP, or with the IC_50_ of cisplatin. Doses of the drugs used for combination treatment are shown in the supplementary (Table S3). All experiments were performed in triplicates and were repeated at least three times.

### *In vitro* trypan blue-based viability assay

The *in vitro* effect of MonA on cell viability was evaluated by trypan blue exclusion assay using semi-automated cell count with a Beckman Coulter Vi-CELL (Beckman Coulter, Krefeld, Germany) as described before [43].

### Protein preparation and western blotting

Preparation of protein extracts and Western blotting were performed as described previously with slight modifications [44]. In brief, 1 × 10^6^ cells/well were seeded in Petri dishes (ø 6 cm, 5 mL/dish). Cells were harvested, proteins were extracted, subjected to electrophoresis in 10-15% SDS-polyacrylamide gels at 120 V, and transferred from gel to a 0.2 μm pore PVDF membrane. The membrane was blocked and incubated with the primary antibody according to the manufacturer's protocol (for antibodies used, see the supplementary). After washing, the membranes were incubated with the corresponding secondary antibody for 1 h at RT. Signals were detected using the ECL chemiluminescence system (Thermo Scientific, Rockford, IL, USA) according to the manufacturer's protocol.

### Staining of the SDS-polyacrylamide gels with Coomassie brilliant blue

SDS-polyacrylamide gels were prepared as described above. Dye-free loading buffer was used for the protein samples preparation. Gels were fixed and stained with colloidal Coomassie Blue overnight, destained, and digitalized as previously described [45].

### Protein identification by mass spectrometry

Protein spots of interest were excised from the gel and digested manually with trypsin. Mass spectrometry measurements and identification were performed as previously described [46]. The peptide solutions were analyzed using a Proxeon nano-LC system equipped with a nano-ESI source (Proxeon, Odense, Denmark) connected to a LTQ-Orbitrap-MS (ThermoElectron, Bremen, Germany). For protein identification, automated database searches using the Sequest algorithm rel. 27.11 (Sorcerer built 4.05, Sage-N Research Inc., Milpitas, CA) were performed using a Swiss-Prot database rel. 2013_10 (limited to human entries). The considered enzyme specificity was fully tryptic. Parent mass tolerance (MS) was set to 10 ppm and fragment mass tolerance 1 Da. Methionine oxidation and propionamid were considered as optional modification. Protein identification was based on a) peptide length of 5, peptide probability: 95.0% minimum and sequest: deltaCn scores of greater than 0.1 and XCorr scores of greater than 2.3, 3.8 and 3.8 for doubly, triply, and quadruply charged peptides and b) protein thresholds: 95.0% minimum and 3 peptides minimum.

### Detection of apoptotic cells by annexin-V-FITC / PI double staining

Induction of apoptosis was examined using FACS-based analysis with an annexin-V-FITC (BD Bioscience, San Jose, CA, USA) and propidium iodide (PI) (Sigma) double staining. The experiment was performed as previously described [47].

### Cell cycle analysis

The cell cycle distribution was analyzed by flow cytometry using PI staining as described before [45] with slight modifications. In brief, cells were pre-incubated overnight in 6-well plates (2 × 10^5^ cells/well). The medium was changed to 0.1% FBS/DMEM medium (DMEM/Glutamax^TM^-I medium containing 0.1% FBS and 1% penicillin/streptomycin). After 24 h of incubation, the medium was changed to normal 10% FBS/DMEM medium containing different concentrations of the substances examined. After 24 h of treatment, cells were harvested with a trypsin-EDTA solution, fixed, stained, and analyzed. The results were quantitatively analyzed using the FlowJo software, v.7 (Treestar Inc, Ashland, OR, USA).

### Detection of activated caspase-3 by FACS

To assess occurrence of caspase-3 cleavage, FACS-based analysis with a cleaved caspase-3 specific, PE-linked antibody (BD Bioscience) was used. NCCIT-R cells were pre-incubated overnight in 6-well plates (2 × 10^5^ cells/well). The medium was replaced by a medium containing different concentrations of the substances examined. After 48 h of treatment, cells were harvested with a trypsin-EDTA solution, washed twice with PBS, and fixated with 2% paraformaldehyde (pH 7.4) for 10 min at 37°C. After incubation on ice for 1 min, cells were pelleted by centrifugation for 5 min at 453 x g, resuspended in 1 mL of 90% methanol, and incubated for 30 min at 4°C. Then 0.5 mL of 0.5% BSA/PBS were added, cells were pelleted (10 min at 453 x g), resuspended in 50 μL of 0.5% BSA/PBS, and incubated for 10 min at RT. 1 μL of either cleaved caspase-3 specific, PE-linked antibody, or PE-linked isotype control, or PBS were added followed by incubation for 60 min at RT in the dark. Afterwards, cells were resuspended in 0.5 mL of 0.5% BSA/PBS, pelleted (10 min at 453 x g), resuspended in 0.3 mL of PBS, and analyzed using the FACS Calibur (BD Bioscience). The results were analyzed using the BD Bioscience Cell Quest Pro software (BD Bioscience).

### Light microscopy

Microphotographs of cells were taken using Axiovert 25 microscope and an AxioCam MRc camera (Carl Zeiss, Göttingen, Germany) at x400 magnification.

### Electronic microscopy

For electron microscopy, untreated and treated NCCIT-R cells were fixed using glutaraldehyde and embedded in Epon-Araldite. Then, semithin and ultrathin sections were cut and analysed using a Zeiss microscope EM 906 (Carl Zeiss, Oberkochen, Germany) at various magnifications.

### Acridine orange staining of lysosomes

NCCIT-R cells were pre-incubated overnight in 8-chamber glass slides (5 × 10^4^ cells/chamber). The medium was changed with medium containing different concentrations of the substances examined, and cells were treated for 60 min. Then cells were washed twice with PBS solution, stained with 0.5 μg/mL of acridine orange/PBS for 15 min at 37°C, covered with DAPI-based ProLong® Gold reagent (Life Technologies, Eugene, OR, USA), and immediately analyzed with AxioScope.A1 (Carl Zeiss) microscope and with the AxioVision40 V4.8 software (Carl Zeiss Imaging Solutions, Göttingen, Germany). Red fluorescence measurements and quantification were performed with Image J software (NIH, Bethesda, USA).

### Detection of cathepsin B release

To assess release of cathepsin B into the extracellular space, NCCIT-R cells were pre-incubated overnight in 6-well plates (2 × 10^5^ cells/well), and were then treated with MonA for 48 h. After trypsination, cells were immobilized on glass slides using the cytospin method and dried overnight. Cells were fixed with 2% (w/v) PFA/PBS, washed with PBS and permeabilized with 0.1% (v/v) Triton X-100 solution in 2% (v/v) normal goat serum/PBS for 30 min. After washing with PBS, samples were treated with 1:400 rabbit anti-cathepsin B antibody solution (in 0.1% (w/v) NaN3; 0.2% (w/v) BSA in PBS, pH 7.4) overnight at 4°C, washed with PBS and incubated with secondary anti-rabbit Alexa Fluor 488-conjugated antibody solution in PSB for 1 h at RT. Then, samples were washed with PBS, covered with DAPI-based ProLong® Gold reagent (Life Technologies) and directly analyzed with AxioScope.A1 (Carl Zeiss) microscope and with the AxioVision40 V4.8 software (Carl Zeiss Imaging Solutions).

### Statistical analysis

Statistical analyses were performed using GraphPad Prism software v. 5.01 (GraphPad Prism software Inc., La Jolla, CA, USA). Data are presented as mean ±SEM (standard error of the mean). All the experiments were performed in triplicates and repeated at least three times. The unpaired Student's t-test was used for comparison of two groups. Differences were considered to be statistically significant if *p* < 0.05.

## SUPPLEMENTARY MATERIAL TABLES


