# Supplementary material for: Marine alkaloid Monanchocidin a overcomes drug resistance by induction of autophagy and lysosomal membrane permeabilization
Source: Oncotarget. 2015 May 19;6(19):17328–41. doi: 10.18632/oncotarget.4175 (PMC4627311; doi:10.18632/oncotarget.4175)
Supplement: Supplementary file 1 [file oncotarget-06-17328-s001.pdf]

Marine alkaloid Monanchocidin A overcomes drug resistance by induction of autophagy and lysosomal membrane permeabilization

Supplementary Material

Table S1. List of antibodies used

Antibodies	Clonality	Source	Cat.-No.	Used concentration	Manufacturer
anti-α-Tubulin	mAb	mouse	T5168	1:5000	Sigma-Aldrich
anti-β-actin	mAb	mouse	sc-47778	1:5000	Santa Cruz
anti-caspase-3	mAb	rabbit	#9665	1:1000	Cell Signaling
anti-Cathepsin B	pAb	rabbit	sc-13985	1:400	Santa Cruz
anti-ERK	mAb	mouse	#9107	1:2000	Cell Signaling
anti-JNK	mAb	rabbit	#9258	1:1000	Cell Signaling
anti-LC3B-I/II	pAb	rabbit	#2775	1:1000	Cell Signaling
anti-p38	mAb	rabbit	#9212	1:1000	Cell Signaling
anti-PARP	pAb	rabbit	#9542	1:1000	Cell Signaling
anti-phospho-ERK	mAb	rabbit	#4377	1:1000	Cell Signaling
anti-phospho-JNK	mAb	rabbit	#4668	1:1000	Cell Signaling
anti-phospho-p38	mAb	rabbit	#4511	1:1000	Cell Signaling
anti-rabbit IgG-Allexa Fluor® 488		goat	#4412	1:1000	Cell Signaling
anti-goat IgG-HRP		rabbit	#31433	1:10000	Thermo Scientific
anti-mouse IgG-HRP		sheep	NXA931	1:10000	GE Healthcare
anti-rabbit IgG-HRP		goat	#7074	1:5000	Cell Signaling
anti-rat IgG-HRP		rabbit	ab6734-1	1:5000	abcam

Cell culture conditions

MRC-9 and HEK 293T cells were cultured in 10% FBS/DMEM medium (DMEM medium supplemented with Glutamax™-I (Invitrogen, Paisley, UK) containing 10% fetal bovine serum (FBS) (Invitrogen) and 1% penicillin/streptomycin (Invitrogen)). PC-3, DU145, TCam-2, RT112, RT4, 486p and T24 cells were cultured in 10% FBS/RPMI medium (RPMI medium supplemented with Glutamax™-I (Invitrogen, Paisley, UK) containing 10% FBS and 1% penicillin/streptomycin). NCCIT, NCCIT-R, 2102EP and 2102EP-R cells were cultured in 10% FBS/DMEM F-12 medium (DMEM F-12 medium (Invitrogen, Paisley, UK) containing 10% FBS, 1% penicillin/streptomycin, and 2 mM glutamine (Invitrogen, Paisley, UK)). LNCaP cells were cultured in 10% FBS/RPMI medium (RPMI medium supplemented with Glutamax™-I containing 10% FBS, 1%

penicillin/streptomycin, and 1 mM sodium pyruvate (Invitrogen, Paisley, UK)). MRC-5 cells were cultured in 10% FBS/MEM medium (MEM medium supplemented with Glutamax^{TM}-I containing 10% FBS, 1% penicillin/streptomycin, and 1 mM sodium pyruvate). HUVEC cells (passage 15) were cultured in Clonetics® EGM^{TM}-2 SingleQuots® medium (Lonza, Walkersville, MD, USA) containing 10% FBS. Cells were incubated at 37°C in a humidified atmosphere with 5% (v/v) CO_2.

Table S2. Identified proteins in the spot appearing in the lower part of the gel in lysates of NCCIT-R cells treated with MonA (identification by LTQ-Orbitrap-MS*)

Sample	Swiss-Prot Accession no	Protein name	Swiss-Prot Accession no	Theor. Mw (kDa)	Number of sequenced peptides	Sequence coverage (%)	Theor. pI
Sample 1	PLAK_HUMAN	Cluster of Junction plakoglobin	P14923	82	9	16	5.8
	PKP1_HUMAN	Isoform 1 of Plakophilin-1	Q13835-2	80	8	13	9.3
	ARGI1_HUMAN	Isoform 2 of Arginase-1	P05089-2	36	6	22	6.7
	TPIS_HUMAN	Isoform 2 of Triephphate isomerase	P60174-1	27	6	29	5.7
	H0YMD0_HUMAN	Cluster of Annexin (Fragment)	H0YMD0	25	5	27	5.7
	SPB3_HUMAN	Isoform 2 of Serpin B3	P29508-2	39	4	15	6.4
	PRDX1_HUMAN	Peroxiredoxin-1	Q06830	22	4	22	8.3
	CYTA_HUMAN	Cystatin-A	P01040	11	3	40	5.4
	G3P_HUMAN	Isoform 2 of Glyceraldehyde-3-phphate dehydrogenase	P04406-2	32	3	15	5.4
	ENOA_HUMAN	Alpha-enolase	P06733	47	3	8,3	8.6
	SPB12_HUMAN	Serpin B12	Q96P63	46	3	10	7.0
	B4DV12_HUMAN	Ubiquitin	B4DV12	17	3	22	5.4
	K2C72_HUMAN	Isoform 2 of Keratin, type II cytkeletal 72	Q14CN4-2	56	3	4,7	6.8
Sample 2	CYTA_HUMAN	Cystatin-A	P01040	11	4	52	5.4
	TPIS_HUMAN	Isoform 2 of Triephphate isomerase	P60174-1	27	3	16	5.7

*Samples numbers are equal to the spot numbers in Figure 3C

Table S3. Concentrations of the substances used for combinational experiments.

Combination	Ratio
MonA : z-VAD-fmk	1.2 µM : 100 µM
MonA : 3-MA	1.2 µM : 5000 µM
MonA : NH_4Cl	1.2 µM : 10000 µM
MonA : calpeptine	1.2 µM : 50 µM
MonA : leupeptine	1.2 µM : 100 µM
MonA : protease inhibitors cocktail (cOmplete Mini EDTA-free)	1.2 µM : 0.02 tablet/ml
